# Factors contributing to long‐term vaginal pessary use: A mixed‐methods study

**DOI:** 10.1002/ijgo.70343

**Published:** 2025-06-30

**Authors:** L. E. Drost, L. Beijleveldt, B. O. Ozéphius, A. Franx, M. C. Vos

**Affiliations:** ^1^ Department of Obstetrics and Gynecology Erasmus Medical Centre Rotterdam The Netherlands; ^2^ Department of Obstetrics and Gynecology Elisabeth Tweesteden Hospital Tilburg The Netherlands

**Keywords:** mixed methods, patient experience, pelvic organ prolapse, pessary, quality of life

## Abstract

**Objective:**

One of the treatment options in pelvic organ prolapse (POP) is vaginal pessary therapy. For a well‐informed decision, different aspects of the treatment option, including sustainable long‐term outcomes, need to be discussed with the patient. However, long‐term quality of life and psychological wellbeing of pessary use is unknown. This study explores the factors contributing to long‐term pessary use.

**Methods:**

A mixed‐methods study was performed among patients with vaginal pessary use for at least 5 years. The quantitative part consisted of online, validated questionnaires on satisfaction with care (Patient Satisfaction Questionnaire Short Form [PSQ‐18]), shared decision‐making (Shared Decision Making Questionnaire [SDM‐Q‐9]) and decisional regret (Decision Regret Scale [DRS]). Health‐related quality of life and pelvic floor complaints were measured by the Pelvic Floor Disability Index (PFDI‐20), Pelvic Floor Impact Questionnaire (PFIQ‐7), Pelvic Organ Prolapse/Urinary Incontinence Sexual Questionnaire (PISQ‐12), and the EuroQol EQ‐5D‐5L questionnaire. The qualitative part comprised semi‐structured interviews according to the Consolidated Criteria for Reporting Qualitative Research (COREQ) analyzed with grounded theory analysis.

**Results:**

In all, 35 patients filled in the questionnaires and seven were interviewed. Overall, patients indicated a good quality of life and satisfaction with the treatment. Three major themes were found in the interviews: effect of the treatment, contact with the healthcare provider, and choice in type of care. Four minor themes were psychosocial aspects, self‐image, established habits, and sexuality.

**Conclusion:**

When considering possible pessary use with a POP patient, these longterm effects of successful pessary treatment can be offered. The identified themes need to be addressed when applicable for the patient. Patients can be informed that long‐term pessary use is feasible and can become a habit with little burden that relieves the complaints they experience.

## 1 INTRODUCTION

Symptomatic pelvic organ prolapse (POP) is a common condition in women, with an estimated prevalence of 11.4% among Dutch women between the age of 45 and 85 years.^1^ It is defined as the descent of at least one of the vaginal walls to or beyond the vaginal hymen in combination with bothersome characteristic symptoms or functional or medical compromise. Characteristic symptoms are vaginal bulge, urinary and defecatory complaints, and sexual dysfunction.^2^ POP can have a far‐reaching impact on the overall state of health and wellbeing and is known to have a significant effect on quality of life.^3^

Treatment options for POP are pelvic floor muscle therapy, surgery, or vaginal pessary therapy. A pessary is a vaginal support device made of silicone or plastic. Pessaries have the potential for long‐term use and avoidance of surgery. They are generally inexpensive, well tolerated, and safe. However, several side effects, such as an increase in vaginal discharge, odor, bleeding, pain, or constipation, have been reported.^4^, ^5^ When considering pessary treatment for POP, these aspects have to be discussed with the patient. Yet women with POP report a lack of information and support which leads to significant anxiety surrounding their diagnosis and treatment, and poor satisfaction.^6^

Previous studies mainly focus on the short‐term improvement of physical symptoms and lack information about the long‐term and psychosocial factors involved in pessary treatment. Several studies focusing on continuation rates show a median duration of use of 3–7 years and continuation rates of 33.5% after 10 years. However, side effects and reasons for discontinuation were captured by chart review, and in‐depth investigation into reasons for discontinuation is lacking.^5^, ^7^, ^8^ In studies that do seek in‐depth explanation, and state that living with a pessary is a life‐changing experience, the average treatment duration was either short or unknown.^9^, ^10^

The aim of this study is to learn more about the factors for continuation of vaginal pessary use for POP in the long term. Next to the physical aspects, the psychological, social, and practical elements will be explored. In a mixed‐methods study design, patients will fill out questionnaires followed by in‐depth interviews for some of them. The acquired knowledge can help to improve future patient counseling concerning pessary treatment and current care for patients with a pessary.

## 2 METHODS

### 2.1 Study design

A mixed‐methods study was performed from October 2021 until October 2023, including a quantitative part consisting of questionnaires and a qualitative part consisting of interviews. The qualitative study design was based on the Consolidated Criteria for Reporting Qualitative Research (COREQ), a checklist for explicit and comprehensive reporting of qualitative studies.^11^ Table 1 shows details of the research methodology according to the COREQ criteria. An embedded design was used, to strengthen the conclusions of the questionnaires with the deeper insights from the interviews. The study was approved by the Medical Ethical Research Committee Brabant, Tilburg, The Netherlands (NW‐2021‐67).

**TABLE 1:** Consolidated Criteria for Reporting Qualitative Research (COREQ).

No	Item	Guide questions/description
**Domain 1: Research team and reflexivity**		
Personal characteristics		
1.	Interviewer/facilitator	LB
2.	Credentials	Research student
3.	Occupation	Medical student at the Erasmus University Rotterdam
4.	Gender	Female
5.	Experience and training	No experience—however, extensive knowledge of interviewing techniques was gained
Relationship with participants		
6.	Relationship established	No relationship
7.	Participant knowledge of the interviewer	Participants were informed about the occupation of the researcher and the aim of the study
8.	Interviewer characteristics	A neutral point of view was maintained
**Domain 2: Study design**		
Theoretical framework		
9.	Methodological orientation and theory	Open‐ended design, grounded theory method
Participant selection		
10.	Sampling	Convenience sampling
11.	Method of approach	By verbal and visual information about the study provided by doctors, messages on the website of patient organizations, and social media
12.	Sample size	Seven patients were included in the qualitative part of this study
13.	Non‐participation	One patient refused to participate for personal reasons
Setting		
14.	Setting of data collection	By telephone
15.	Presence of non‐participants	No other persons were present during the interviews
16.	Description of sample	Women who used a vaginal pessary as a treatment for POP for at least 5 years
Data collection		
17.	Interview guide	A semi‐structured interview guide was developed by the research team and reviewed by the patient organization and an uninvolved gynecologist
18.	Repeat interviews	No repeat interviews were carried out
19.	Audio/visual recording	Audio of all interviews was recorded by the application TapeACall
20.	Field notes	Field notes were made during the interviews to indicate key points
21.	Duration	The duration of the interviews was between 20 and 47 minutes
22.	Data saturation	Data saturation was reached after five interviews
23.	Transcripts returned	Transcripts were not returned to participants for comment and/or correction
**Domain 3: Analysis and findings**		
Data analysis		
24.	Number of data coders	Data were coded by two researchers independently (LB and BO)
25.	Description of the coding tree	An extensive description of the coding tree can be provided on request
26.	Derivation of themes	Themes were gradually derived during the coding process
27.	Software	Atlas.ti was used to manage the data
28.	Participant checking	Participants did not provide feedback on the findings
Reporting		
29.	Quotations presented	Participant quotations were presented to illustrate the themes/findings and each quotation was identified
30.	Data and findings consistent	There was consistency between the data presented and the findings
31.	Clarity of major themes	Major themes are presented in Table 5 and explained in text, including quotes
32.	Clarity of minor themes	Minor themes are presented in Table 5 and explained in text, including quotes

Abbreviation: POP, pelvic organ prolapse.

### 2.2 Participants

Women who used a vaginal pessary as a treatment for POP for at least 5 years were eligible for participation. Furthermore, participants had to have sufficient understanding of the Dutch language to fill out the questionnaires. Convenience sampling was used, as the target population does not necessarily visit a healthcare provider regularly. Participants were recruited through verbal and visual information about the study provided by several gynecologists and general practitioners, messages on the website of the Dutch patient organizations Bekkenbodem4all and Vuurvrouw, and social media. All patients were requested to fill out the questionnaires for the quantitative part of the study and, in addition, patients were invited for an interview. Written informed consent was obtained for all participants.

### 2.3 Data collection

For the quantitative part of the study, patients were provided validated questionnaires online at one point in time. The questionnaires concerned satisfaction with shared decision‐making (Shared Decision Making Questionnaire [SDM‐Q‐9]), satisfaction with care (Patient Satisfaction Questionnaire Short Form [PSQ‐18]), decisional regret (Decision Regret Scale [DRS]) and anxiety and depression (Hospital Anxiety and Depression Scale [HADS]).^12^, ^13^, ^14^, ^15^ Furthermore, health‐related quality of life and complaints of the pelvic floor were measured by the Pelvic Floor Disability Index (PFDI‐20), Pelvic Floor Impact Questionnaire (PFIQ‐7), Pelvic Organ Prolapse/Urinary Incontinence Sexual Questionnaire (PISQ‐12) and the EuroQol EQ‐5D‐5L questionnaire.^16^, ^17^, ^18^ All questionnaires were distributed through PROFILES (Tilburg, the Netherlands) and consisted of four‐ to six‐item Likert‐scales. Age, body mass index (BMI, calculated as weight in kilograms divided by the square of height in meters), educational level, smoking history, and obstetric history were collected separately for all patients.

For the qualitative part of the study, individual interviews were conducted using a semi‐structured interview guide. The interview guide was developed by the research team and reviewed by the patient organization, Bekkenbodem4all, and a gynecologist who had not been involved in the study. Topics discussed in this guide are the organization of care for women with a pessary, symptoms and side effects, psychosocial aspects of a pessary, and sexuality with a pessary. Open‐ended questions were used to encourage the participants to speak openly. Additions to the interview guide were made if new questions were considered relevant after an interview. The interviews were conducted face‐to‐face or by phone by one researcher and took approximately 30 min. All interviews were recorded and transcribed verbatim.

### 2.4 Data analysis

For the quantitative part of the study, data were analyzed using SPSS version 28 (IBM, Armonk, NY, USA). Descriptive statistics were used to give an overview of the sociodemographic data and the questionnaires. No statistical tests concerning any statistical significance were used.

For the qualitative part of the study, grounded theory analysis was performed. A first version of the coding scheme was drafted while transcribing the interviews. With this codebook, the discussed topics were coded line‐by‐line with the software program ATLAS.ti (ATLAS.ti Scientific Software Development GmbH, Berlin, Germany). If necessary, adjustments to the coding scheme were made during the coding process. For analysis the coded phrases were grouped into higher‐order themes. Each coded interview was revised by two researchers (LB and BO) and, by consultation between them, agreement upon the applied codes was reached. The final coding scheme, a non‐hierarchical concept list, is given in Table 2. Illustrative quotes were translated from Dutch to English.

**TABLE 2:** Coding scheme: Non‐hierarchical concept list.

Shared decision‐making
Experiences
Expectations
Importance
Care and control of vaginal pessary
Experiences
Patient preferences
Reasons for preferences
Independence
In care and cleaning
Dealing with side effects
Symptoms and improvement
Experiences
Satisfaction
Side effects of vaginal pessary
Experienced complaints
Solutions
Expectations
Causality
Possibility to discuss with caregivers
Experiences
Possible barriers
Importance
Possibility to discuss with family and friends and experience with taboo
Possible barriers
Importance
Self‐image
Impact of the pessary
Impact of the symptoms
Anxious or depressive thoughts
Negative emotions
Severity
Established habits
Rituals
Integration in daily life
Influence of pessary
Experiences
Solutions
Potential to discuss with sexual partner
Barriers

## 3 RESULTS

In all, 85 patients who had been using a pessary for POP for at least 5 years were eligible for inclusion in the study; 35 patients, who signed informed consent and filled out the questionnaires, were included in the quantitative part of this study. Additionally, seven patients were interviewed. The average age of the participants was 72.5 years. Patient characteristics are given in Table 3. All interviews were done by a researcher (LB) who was not involved in the care for the patients but only conducted the interviews. Patients were interviewed by telephone and were in a place of their preference. No new themes appeared in the last two interviews, indicating data saturation. For this reason, not all patients who filled out the questionnaires were interviewed.

**TABLE 3:** Patient characteristics.

	Quantitative study (*n* = 35)	Qualitative study (*n* = 7)
Age (y) (mean [SD])	72.5 (10.0)	69.9 (10.2)
BMI (mean [SD])	25.1 (3.12)	25.0 (1.50)
Parity (mean [SD])	2.35 (0.71)	2.14 (0.38)
Educational level (*n* [%])		
Primary school	2 (9.1%)	0 (0%)
Secondary school	7 (31.8%)	0 (0%)
Secondary vocational education	4 (18.2%)	2 (33.3%)
Higher professional education	7 (31.8%)	3 (50%)
University education	2 (9.1%)	1 (16.7%)
Unknown	13	1
Smoking (*n* [%])		
Yes	1 (4.3%)	0 (0%)
No	22 (95.7%)	6 (100%)
Unknown	12	1

### 3.1 Questionnaires

On the EQ‐5D‐5L, the average index score was 0.92 on a scale of −0.59 to 1, where 1 indicates the best health one can imagine; 18 patients (51.4%) reported a value of 1.^16^ Scores and subscale scores of all questionnaires are shown in Table 4.

**TABLE 4:** Results of questionnaires.

Questionnaire	Range of scores	Mean (SD)
EQ‐5D‐5L	5–25	6.11 (1.69)
EQ‐5D‐5L index	−0.59–1	0.92 (0.09)
EQ‐VAS	0–100	81.2 (17.7)
PFDI‐20 (pelvic floor disability)	0–300	60.1 (52.7)
POPDI‐6 (pelvic organ prolapse distress)	0–100	17.1 (24.0)
CRADI‐8 (colorectal‐anal distress)	0–100	15.8 (17.8)
UDI‐6 (urinary distress)	0–100	27.1 (20.1)
PFIQ‐7 (pelvic floor impact)	0–300	27.8 (33.0)
UIQ‐7 (urinary impact)	0–100	11.7 (14.3)
CRAIQ‐7 (colorectal‐anal impact)	0–100	11.2 (20.0)
POPIQ‐7 (pelvic organ prolapse impact)	0–100	4.90 (8.37)
SDM‐Q‐9 (shared decision‐making)	0–45	32.9 (11.1)
SDM‐Q‐9 rescaled	0–100	73.1 (24.7)
PSQ‐18 (patient satisfaction)	18–90	71.3 (12.4)
General satisfaction	1–5	3.99 (0.82)
Technical quality	1–5	3.94 (0.78)
Interpersonal manner	1–5	4.24 (0.75)
Communication	1–5	3.94 (0.84)
Financial aspects	1–5	4.07 (0.77)
Time spent with doctor	1–5	3.91 (0.90)
Accessibility and convenience	1–5	3.81 (0.72)
DRS (decisional regret)	0–100	15.4 (18.0)
HADS (anxiety and depression)	0–42	6.94 (4.93)
Anxiety	0–21	4.34 (3.11)
Depression	0–21	2.60 (2.26)
PISQ‐12 (sexual functioning)	0–48	33.5 (5.76)
Behavioral‐emotive	0–16	10.0 (3.30)
Physical	0–20	16.3 (3.45)
Partner‐related	0–12	7.25 (2.31)

Abbreviations: DRS, Decision Regret Scale; EQ‐5D‐5L, EuroQol EQ‐5D‐5L; HADS, Hospital Anxiety and Depression Scale; PFDI‐20, Pelvic Floor Disability Index; PFIQ‐7, Pelvic Floor Impact Questionnaire; PISQ‐12, Pelvic Organ Prolapse/Urinary Incontinence Sexual Questionnaire; PSQ‐18, Patient Satisfaction Questionnaire Short Form; SDM‐Q‐9, Shared Decision Making Questionnaire.

The average score on the PFDI‐20 was 60.1 on a scale of 0–300, where higher values indicate greater distress caused by the presence of pelvic floor symptoms. The average score on the PFIQ‐7 was 27.8 out of 300, with a higher score meaning there is a higher effect of symptoms on quality of life.^17^

On the SDM‐Q9, patients scored an average total of 32.9 on a scale of 0–45, with 0 indicating the lowest and 45 indicating the highest level of perceived shared decision‐making.^12^ On the PSQ‐18, the average score was 71.3 on a scale of 18–90, where 90 indicates the highest patient satisfaction.^14^

On the DRS, patients scored an average of 15.4 on a scale of 0–100, a higher number indicating more regret—29 patients (82.8%) agreed or strongly agreed that they had made the right decision by choosing pessary treatment over 5 years ago and 29 patients (82.8%) would go for the same choice if they had to do it over again. Only 1 patient (2.9%) regretted the choice that was made and 4 patients (11.4%) would not choose the same option if they had to do it over again.^15^

On the HADS questionnaire, the average score was 6.94 on a scale of 0–42. Looking at the anxiety disorder subscale, 3 patients (8.6%) scored between 8 and 10, indicating a possible anxiety disorder, and 2 patients (5.8%) scored 11 or higher, indicating a probable anxiety disorder. On the depression subscale all patients scored 7 or lower, indicating no signs of depression.^13^

Eight patients (22.9%) indicated that they were sexually active and filled out the PISQ‐12 questionnaire; 22 patients (62.9%) were not sexually active, and 5 patients (14.3%) did not want to answer the question. The average score on the PISQ‐12 questionnaire was 33.5 on a scale of 0–42, where higher scores indicate better sexual function.

### 3.2 Interviews

Three major themes were found: effect of the treatment, contact with the healthcare provider, and choice of type of care. Furthermore, four minor themes appeared: psychosocial aspects, self‐image, established habits, and sexuality. An overview of the themes is presented in Table 5.

**TABLE 5:** Overview of themes.

Themes	Frequency (*N* = 168)
Treatment effect	
Symptoms and improvement	23
Side effects	22
Contact with healthcare provider	
Possibility to discuss pessary use with healthcare providers	20
Shared decision‐making	19
Type of care	
Care and control	22
Independence	8
Psychosocial aspects	
Possibility to discuss with family and friends/experienced taboo	15
Anxious or depressive thoughts	9
Self‐image	10
Established habits	10
Sexuality	
Influence of pessary on sexuality	8
Possibility to discuss with sex partner	2

#### 3.2.1 Treatment effect

Patients were generally satisfied with the use of the pessary. They all experienced side effects to some extent, such as vaginal dryness, vaginal discharge, irritation of the vaginal mucosa, difficulty with urination, or obstipation. However, they were generally not bothered by those side effects as the pessary solved a major health issue for them.

A very easy way to get rid of a major ailment. (Patient 3)

I rub it with Zwitsal® and it's done. (Patient 1)

#### 3.2.2 Contact with healthcare provider

During the interviews several patients posed questions about prolapse and pessary use. Those questions concerned a possible indication for surgery but also whether a pessary has an expiration date or if there were tools for easier removal. When clarifying whether they addressed those questions to a healthcare provider, it became clear that the patients who do self‐management of the pessary instead of regular visits to a healthcare provider for pessary care, experience a barrier to seek clinical care. They did not want to disturb a clinician with a question that was, according to their own opinion, non‐urgent.

Questions come to my mind once in a while, but not urgent enough to contact my general practitioner. (Patient 6)

One patient specifically explained that an annual contact would be enough to answer sporadic questions and that it would give her a safe feeling about the pessary use. The relationship she would build with the clinician would remove the barrier to ask questions.

When remembering the initial treatment decision, patients were satisfied about the way the decision was made in the first place. Six patients had the feeling that the decision was their own and that they were fully informed by the healthcare professional.

We talked about the pessary and also about other options … We had a good and extensive talk and I was helped very well. (Patient 1)

#### 3.2.3 Type of care

There were two opposing views on care preferences around pessary treatment. Four women preferred self‐management of the pessary and said they would not prefer care from a healthcare professional. They preferred to remove, clean and re‐insert the pessary themselves at home. Different reasons were mentioned for this, such as a financial stimulus or the burden on the healthcare system. All four of them mentioned independence as a factor for their satisfaction with the pessary. It saved time, felt cleaner, and avoided an intimate consultation with a healthcare provider. Two of them removed the pessary daily before going to sleep and placed it back in the morning. The other two patients cleaned the pessary by themselves less frequently.

A friend said: I do that myself. Then I thought: I can do that too. (Patient 2)

On the other hand, three of the interviewed women were more comfortable with visiting a general practitioner or gynecologist periodically. Reasons for this were uncertainty and discomfort with changing the pessary themselves or the safe feeling of being checked by an expert.

My general practitioner is very kind and says that I can also do it myself, but I just don't feel comfortable with it. (Patient 3)

#### 3.2.4 Psychosocial aspects

The taboo around this subject was recognized by all interviewed patients. Three patients felt uncomfortable talking about it freely. The other four patients had satisfying conversations with close family and friends.

It's not something you just throw on the table over coffee. (Patient 6)

Six patients never felt anxious or depressed about using the pessary. Two of them mentioned that they experienced mild negative emotions about it. One patient did not like the pessary because she experienced a lot of side effects and these sometimes held her back from daily activities.

I don't have strong emotions about it, but it does bother me sometimes. (Patient 2)

#### 3.2.5 Self‐image

Five patients mentioned that using a pessary did not change their self‐image. In fact, three of them specifically mentioned that their self‐esteem had improved since the start of the therapy.

I would feel very differently if I did not have it (the pessary). I would feel the prolapse all the time, that would make me feel worse than I feel now. Now it's solved. (Patient 1)

However, the prolapse itself or the urine incontinence that they experienced did impact the self‐image of three patients. They found it confronting because they considered their pessary as a sign of aging, or they felt insecure about it during sexual activities.

When you're losing urine every now and then, you're afraid that people see or smell it. That has an impact on your self‐image. (Patient 2)

#### 3.2.6 Established habits

Six patients in this study experienced the use of the pessary as a habit. These were both patients who visited a clinician on a regular basis as well as patients who took care of the pessary by themselves. Most of the time they forgot about having a pessary or they saw it as part of their daily life.

A ritual has emerged which is part of my daily care. (Patient 2)

It's like flossing your teeth, it's just part of it you know. (Patient 7)

The one patient who did not experience the use of the pessary as a daily habit was bothered by side effects and was frequently occupied with thoughts about the pessary.

#### 3.2.7 Sexuality

Three women were still sexually active on a regular basis and four women were not sexually active due to reasons other than the pessary. Two women reported that the pessary created insecurity about sexual activity in the beginning. Initially, they did not know if and how the pessary would affect their sex life. They eventually found answers to these questions by trying it out themselves.

We thought; is that even possible? Won't it (the pessary) move or something? (Patient 3)

One patient expressed the need for better information provision concerning sexuality, whereas another patient would have preferred not to discuss sexuality with her gynecologist.

## 4 DISCUSSION

The present study offers insights into the experiences of women with long‐term pessary use. Using a mixed‐methods design, daily functioning and patient satisfaction were measured with questionnaires, followed by in‐depth interviews with the first seven patients included in the study. Patients rated their quality of life as good with a low impact of pelvic floor symptoms in daily life. Patient satisfaction was high with low rates of decisional regret, anxiety, and depression. In the qualitative part, three major themes were found: effect of the treatment, contact with the healthcare provider, and choice of type of care. Four minor themes were identified: psychosocial aspects, self‐image, established habits, and sexuality.

In the questionnaires as well as in the interviews, patients mentioned their satisfaction with pessary treatment and the fact that it had a low impact on daily life. A recent Dutch study by Van der Vaart et al., which followed women with a pessary over 2 years, showed slightly lower PFDI‐20 and PFIQ‐7 scores, revealing less impact of pelvic floor symptoms in daily life than was reported by our patients.^19^ Other studies assessing symptoms also had a shorter follow‐up period than our study and showed slightly lower scores, except for a recent study by Hagen et al. who reported moderately higher scores after 18 months of pessary use, depending on the type of care.^20^, ^21^, ^22^ The study of Hagen et al. also showed a slightly worse quality of life than was found in our population.^22^ Nonetheless, as we have collected unique long‐term data, our results cannot be compared directly with previous research, due to the difference in the follow‐up period.

When reflecting on the initial contact with the healthcare professional, patients were still satisfied with shared decision‐making. This was shown by the high score on the SDM‐Q‐9 and confirmed in the interviews. Yet patients mentioned that pessary use sporadically raised questions, even after long‐term use. They felt a certain barrier to contacting a healthcare professional after a long period without consultations. This could also be inferred from the PSQ‐18, where the domain ‘accessibility and convenience’ scores were lower compared with the other subscales. We believe that these barriers should be reduced, as resolving small side effects or questions will result in more satisfactory and longer pessary use. Previous research described that visiting a clinic helps patients bond with clinicians and that they feel emotionally supported as a result.^10^ The barriers preventing patients from seeking help differed widely between patients and had not been studied before in POP patients during pessary treatment.

Concerning the third major theme, care and control of a pessary, two groups emerged: some patients felt more comfortable with periodically visiting a healthcare professional for pessary care and some patients preferred to take care of the pessary by themselves, independently of a healthcare provider. This dichotomy was also seen in a Canadian study focusing on the experiences of women using a pessary for urinary incontinence or POP.^10^ In that study, age and confidence in care were stated as factors withholding women from self‐care. Those reasons also emerged in the present study. However, non‐medical reasons for self‐care were not mentioned in earlier research. Hagen et al. showed that pessary self‐management has a lower complication rate, does not influence quality of life compared with clinic‐based care, and is cost‐effective.^22^ Paulussen et al. also showed a lower rate of treatment discontinuation in women performing pessary self‐management.^23^ It is also known to result in greater confidence in the management of pessary problems and freedom to choose when to use the pessary or not.^10^, ^22^

Concerning the minor themes, the majority of the patients mentioned in the interviews that they never felt anxious or depressed about their pessary use. This was confirmed by the low HADS questionnaire score. A study by Kalata et al. showed a correlation between POP and depression, anxiety, and insomnia.^24^ As different questionnaires were used and the control group consisted of patients with other gynecological problems in the same center, results are hard to compare.

In our study, patients also mentioned that the pessary itself does not have a negative effect on their self‐esteem. However, the presence of the prolapse and urine incontinence did have an effect. Even though previously performed studies showed that urine incontinence and POP negatively affect self‐image, this has never been studied in relation to POP and pessary use specifically.^25^, ^26^ As a pessary can also resolve incontinence problems, it is likely to have a positive effect on women's self‐esteem.

Also, body image during sexual activities changed for some women when they started to use the pessary. It took time for patients to work out what was possible and feel comfortable during sexual activity with a pessary. A study by Meriwether et al. showed that a pessary for POP or urinary incontinence did not affect sexual function scores 3 months after the start of pessary treatment in sexually active women.^27^ As a different version of the PISQ was used in this study, it is not possible to compare the results exactly. The PEOPLE study showed that pessary therapy positively affects the impact of POP on sexual activity after 24 months, as measured by the condition‐impact domain of the PISQ.^28^ This is comparable to our results, as women mentioned that it took some time to explore possibilities and gain confidence. A literature review concluded that sexual function should be assessed and discussed as part of routine care in women with a pessary.^29^

Concerning the last minor theme of habit formation, it was found that patients often experience the pessary use as a habit after more than 5 years. Side effects and slight discomfort were reported, but almost all women easily found a way to solve these problems. As other long‐term follow‐up studies concerning pessary use only consisted of retrospective chart reviews, this has never been reported before.^5^, ^8^

A common limitation in qualitative research is researcher bias. The interviews were performed by one person (LB) and depended partially on this researcher's perspective. To minimalize subjectivity, each interview and its coding were reviewed by one or two researchers (LB and BO). Furthermore, the identity and background of the researchers are revealed, in accordance with the COREQ criteria, to provide transparency in the coding process.^11^ As master's students, they had background knowledge but were not involved in the treatment of the patients. Also, there is likely to have been some selection bias due to the method we used for the recruitment of patients. Initially the questionnaires were only provided online and we noticed that for some women it was difficult to complete them. This could have been due to the older age and (digital) health literacy of the study population. As soon as we discovered this, we provided paper versions of the questionnaires for patients. Another possible limitation was the inability to observe non‐verbal communication during a telephone interview. As the study took place during the COVID‐19 pandemic, it was not possible to have face‐to‐face conversations. This deficit has been overcome by deliberately verifying patients' responses. Lastly, collecting information on the specific type of pessary used and the exact frequency of pessary changes could have been of added value, as it would have offered better guidance for healthcare providers and future patients. However, such in‐depth data did not emerge from all interviews.

## 5 CONCLUSION

Several factors concerning long‐term pessary use were found in this study, which can be used to improve information provision, shared decision‐making, and care for patients who opt to use a pessary in the long term. It is important to provide care adapted to the needs of the specific patient. Hence, routine appointments should be planned or adequate instructions for self‐care should be provided, combined with instructions on how to seek contact when necessary. Patients should be made aware of the potential side effects on self‐esteem and the formation of habits concerning the pessary. In addition, every patient should be asked if information on sexuality is preferred. Further research is needed to examine whether providing information on long‐term pessary use to patients who are choosing treatment for POP results in increased patient satisfaction and longer pessary use. Moreover, research into the observed barriers faced by patients in consulting healthcare providers might improve satisfaction with and duration of pessary treatment.

## AUTHOR CONTRIBUTIONS

LED: conceptualization, methodology, formal analysis, project administration, validation, writing—original draft preparation (lead). LB: methodology, investigation, formal analysis, validation, writing—original draft preparation (supporting). BOO: methodology, investigation, formal analysis, validation, writing—review and editing. AF: conceptualization, methodology, supervision, writing—review and editing. MCV: conceptualization, methodology, project administration, supervision, writing—review and editing.

## FUNDING INFORMATION

None.

## CONFLICT OF INTEREST STATEMENT

The authors have no conflicts of interest.

## Data Availability

The data that support the findings of this study are available from the corresponding author upon reasonable request.
